# Olfactory dysfunction with traumatic brain injury and posttraumatic-stress symptoms in post-deployed military personnel

**DOI:** 10.3389/fneur.2025.1569003

**Published:** 2025-10-07

**Authors:** Robert D. Shura, Treven C. Pickett, Jacob Powell, Ruth Yoash-Gantz, Scott D. McDonald, William C. Walker, Jared A. Rowland, Larry A. Tupler

**Affiliations:** ^1^Neurocognition Laboratory, Department of Veterans Affairs Mid-Atlantic Mental Illness Research, Education, and Clinical Center (MA-MIRECC), Durham, NC, United States; ^2^Salisbury Veterans Affairs Health Care System (SVAHCS), Salisbury, NC, United States; ^3^Department of Neurology, Wake Forest University, Winston-Salem, NC, United States; ^4^Department of Physical Medicine and Rehabilitation, Virginia Commonwealth University School of Medicine, Richmond, VA, United States; ^5^National Intrepid Center of Excellence (NICoE), Walter Reed National Military Medical Center, Bethesda, MD, United States; ^6^Richmond Veterans Affairs (VA) Medical Center, Central Virginia VA Health Care System, Richmond, VA, United States; ^7^Department of Psychology, Virginia Commonwealth University, Richmond, VA, United States; ^8^Department of Translational Neuroscience, Wake Forest University, Winston-Salem, NC, United States; ^9^Biodemography of Aging Research Unit, Center for Population Health and Aging, Duke University, Durham, NC, United States; ^10^Department of Psychiatry and Behavioral Sciences, Duke University School of Medicine, Durham, NC, United States

**Keywords:** olfaction, posttraumatic stress disorder (PTSD), traumatic brain injury (TBI), veteran, service member

## Abstract

**Introduction:**

Prior research suggests that olfactory dysfunction may occur following a traumatic brain injury (TBI) due to structural injury to the olfactory peripheral or central networks. Olfaction may also be affected in posttraumatic stress disorder (PTSD) due to traumatic re-experiencing. Given the relevance of both TBI and PTSD to the military and veteran populations, the purpose of this study was to evaluate whether the University of Pennsylvania Smell Identification Test (UPSIT) would be useful in differentiating TBI from significant PTSD symptom burden in a sample of post-deployed active-duty military and veterans.

**Methods:**

A sample of 276 participants with UPSIT data and passing scores on validity measures completed a larger study on neurocognition of predominantly post-deployed veterans of the wars in Afghanistan and Iraq. TBI history was ascertained by medical records or a self-report questionnaire; PTSD symptoms were measured using the PTSD Checklist-Military version (PCL-M) and the Traumatic Stress scale (ARD-T) of the Personality Assessment Inventory. Those with a history of TBI (+TBI) were compared with those without (–TBI) on total UPSIT score; severity of injury and number of injuries were also evaluated. Furthermore, those with and without significant PTSD symptoms (+PTSD and –PTSD) were compared on UPSIT total scores. Finally, group comparisons were conducted to assess whether PTSD demonstrated a significant effect above and beyond TBI.

**Results:**

History of TBI was associated with lower UPSIT scores (–TBI *M* = 34.02, +TBI *M* = 32.76, *z* = −2.38, *p* = 0.017, *r* = 0.14); however, the effect size was small and driven by the difference between moderate/severe TBI and –TBI (moderate/severe *M* = 31.78). Number of mild TBIs was not associated with UPSIT scores: The presence of PTSD symptoms and symptom clusters were not significantly associated with UPSIT scores. PTSD symptoms showed no additional effects on poorer olfaction scores above and beyond TBI.

**Discussion:**

Olfactory identification was significantly reduced in those with a history of TBI, suggesting that olfaction may be useful in the assessment of these individuals for potential treatment needs. Veterans with significant PTSD symptoms, however, did not display different olfactory ability compared with those without, regardless of TBI status.

## Introduction

The recent conflicts in Afghanistan (Operation Enduring Freedom; OEF) and Iraq (Operation Iraqi Freedom; OIF) are associated with large numbers of “signature injuries,” notably traumatic brain injury (TBI) and posttraumatic stress disorder (PTSD) (1–3). These conditions present differential diagnostic challenges due to overlapping symptomatology (4), a complexity compounded in cases of mild TBI (mTBI) or “concussion,” where distinguishing between neurological and psychological symptoms can present marked specificity issues. Determining the etiology of post-concussive or posttraumatic symptoms is important because of different treatment strategies. More precise differential diagnostic tools that clearly outline symptom contributions may facilitate more informed and patient-centered treatment planning. Whereas existent research has focused on cognitive or psychological impairment in those with a history or TBI or PTSD, olfaction may show promise as an additional target in the assessment of these conditions. The purpose of this paper is to evaluate olfactory ability in a sample of veterans, with whom both TBI and PTSD are common diagnostic foci.

Given the evolutionary significance and extensive genetic dedication to olfactory senses in mammals, olfactory examination may offer a way to better discern the relative contribution of post-concussive or posttraumatic symptoms. Olfactory impairments such as anosmia and dysosmia significantly diminish quality of life and are frequently associated with TBI (5–12). Olfaction is also implicated in PTSD-related re-experiencing, underscoring the potential of its assessment in helping to differentiate symptoms of TBI from PTSD (13). Diagnostic tools for assessing the relative involvement of TBI versus PTSD symptom presentation are needed to inform clinical decision making and patient care.

Olfactory neuroanatomy is characterized by connections to the orbitofrontal cortex and limbic structures. Damage to these connections, as may occur secondary to blunt-force trauma in TBI, can lead to a range of olfactory dysfunctions (10, 14, 15). These dysfunctions may be protracted, with severity correlating with the extent of TBI (16–18). Olfactory impairments in PTSD, while less studied, suggest possible diagnostic utility when evaluating responses to specific odorants (19). Previous research indicates that individuals with PTSD exhibit distinct olfactory-identification patterns, especially with odors related to traumatic experiences (13, 17, 20). The present research aims to determine whether olfactory testing, a noninvasive and cost-effective method to assess disruption in the ability to identify odorants more broadly, adds to diagnostic discrimination sensitivity and specificity in TBI and PTSD (11, 17, 19, 21).

The purpose of this study was to evaluate olfaction across TBI and PTSD. We examined four groups: current and former military post-deployed participants with a TBI history (+TBI, any severity); participants with a substantial level of *Diagnostic and Statistical Manual of Mental Disorders–Fourth Edition, Text Revision* (*DSM-IV-TR*) PTSD-symptom burden (+PTSD); and the intersectionality of the two presentations, including participants with both (+TBI/+PTSD) and control participants with neither presentation (–TBI/–PTSD). Olfactory performance is operationalized using the University of Pennsylvania Smell Identification Test (UPSIT), a reliable and valid instrument frequently employed in prior studies of TBI and PTSD (18, 19, 22–27). We hypothesized the following: (H1) Participants with a history of TBI (+TBI) will show significantly poorer olfactory-identification performance than participants negative for such history (–TBI). (H2) Olfaction performance will be negatively associated with higher severity of TBI. (H3) Participants in the +PTSD group will show significantly poorer olfaction performance than those in the –PTSD group. (H4) PTSD will have an interactive effect on olfactory deficits for participants with a history of TBI (i.e., +TBI/+PTSD will show greater deficits than TBI alone (+TBI/–PTSD).

## Materials and methods

### Participants

Data were acquired from 428 active-duty and veteran personnel of the U.S. Armed Forces who completed an extensive neuropsychological battery conducted by the Neurocognition Laboratory of the Department of Veterans Affairs (VA) Mid-Atlantic Research, Education, and Clinical Center (MA-MIRECC) (28). The current analyses are secondary analyses to the study’s main aims, which was to investigate cognitive differences across TBI and PTSD. Those missing UPSIT or validity-test data were excluded (*n* = 35), leaving a working sample of 393 participants. The final sample was *N* = 276 after excluding those with invalid scores on performance- and symptom-validity measures. Recruitment and testing were performed at three VA Medical Center (VAMC) sites, with valid participants in the working sample from the Central Virginia VA Health Care System in Richmond, Virginia (*n* = 36); Durham VAMC in Durham, NC (*n* = 41); and Salisbury Veterans Affairs Health Care System in Salisbury, NC (*n* = 199). Participants were recruited from the MA-MIRECC Post-Deployment Mental Health (PDMH) subject registry (28), the Richmond VAMC Polytrauma Rehabilitation Center (PRC) and Polytrauma Network Site (PNS), and the Durham and Salisbury Level III Polytrauma Support Clinical Team (PSCT) sites. Institutional Review Board approval was obtained separately at each facility. Volunteers were paid $150 plus travel expenses for participation. Inclusion criteria required service in the Armed Forces on or after September 11, 2001. Exclusion criteria consisted of evidence of primary language other than English, difficulty comprehending the informed-consent form or process, psychosis, and/or current substance abuse or dependence as per criteria of *DSM-IV-TR*. Demographics and characteristics for the final, valid sample are presented in Table 1.

**Table 1:** Sample demographics and characteristics (*N* = 276).

Variable	*M* (SD; min–max) or *N* (%)	TBI *n* = 105	No TBI *n* = 171	*p*	PTSD *N* = 107	No PTSD *N* = 169	*p*
Age							
Years	35.96 (10.03; 19–64)	33.70 (9.88)	37.35 (9.90)	<0.001	35.05 (9.23)	36.54 (10.49)	0.305
Race							
White	194 (70.29%)	84 (80.0%)	110 (64.3%)	0.006	65 (60.7%)	129 (76.3%)	0.006
Black	70 (25.36%)						
Other	12 (4.35%)						
Ethnicity							
Hispanic	10 (3.62%)	6 (5.7%)	4 (2.3%)	0.187	6 (5.6%)	4 (2.4%)	0.193
Non-Hispanic	266 (96.38%)						
Sex							
Male	235 (85.14%)	92 (87.6%)	143 (83.6%)	0.820	89 (83.2%)	146 (86.4%)	0.465
Female	41 (14.86%)						
Education							
Years	14.18 (1.91; 11–20)	13.76 (1.84)	14.44 (1.92)	0.003	13.66 (1.65)	14.51 (2.00)	<0.001
TBI							
Mild	69 (25.00%)						
Moderate/severe	36 (13.04%)						

TBI, traumatic brain injury, history of any injury event; PTSD, posttraumatic stress disorder, Positive is endorsement of significant symptoms per the PTSD Checklist-Military version or the Personality Assessment Inventory. Fisher’s Exact Test used for ethnicity comparisons.

### Measures

Olfactory identification was operationalized using the UPSIT (Sensonics Inc., Haddon Heights, NJ, USA) (29). The UPSIT contains 40 multiple-choice items presented in four booklets of 10 items each in which the participant uses a pencil to scratch a 1″ X 0.5″ round-edged rectangular sandpaper odor strip that releases microencapsulated chemosensory stimuli. The participant then places the booklet under his/her nose, sniffs the sandpaper rectangle, and fills in one of four bubbles labeled with the correct odor and three incorrect distractor items randomly ordered across the 40 odorants. The operational measure consists of the number of correct items identified out of 40. Correlations between performance on the four test booklets range between 0.73 and 0.78 (29). Split-half reliability using the Spearman–Brown formula is 0.93 (30). Test–retest reliability ranges from 0.95 when tested 2 weeks apart (31) to 0.92 when tested 6 or more months apart (22).

History of TBI was ascertained from medical records for participants recruited from the Richmond Polytrauma programs and from self-report for those selected from the PDMH subject registry. From medical records, level of consciousness was recorded as intact, becoming “dazed or confused,” or sustaining loss of consciousness (LOC) for a specific duration immediately post trauma. Self-report from PDMH participants was obtained using the Ivins TBI Screen (32). This self-report questionnaire assesses a history of head injury and how many events were experienced, followed by six items which evaluate the presence of alteration of consciousness after the injury, presence and duration of posttraumatic amnesia, and presence and duration of loss of consciousness for each event. Prior work has utilized this screening instrument to establish rates of TBI in paratroopers compared with other military occupational specialties at the largest U.S. Army base, Fort Bragg, located in regional proximity to the two NC sites in the current study (32). Severity of TBI (mild vs. moderate or severe) was based on self-reported LOC (<20 min as mild) and PTA (<24 h as mild) from the time immediately after the injury.

Performance validity was assessed using the Word Memory Test (33), a well-validated, memory-based validity measure. Cutoff scores were used based on the test manual. Symptom validity was assessed via PAI scales (see below) and the Miller Forensic Assessment of Symptoms Test (34), which is a 25-item symptom-validity measure administered in interview format. The cutoff score recommended in the manual was used.

PTSD symptom burden was operationalized using the PCL-M (35–37) and the ARD-T score from the Personality Assessment Inventory. The PCL-M contains 17 items assessing the extent to which the respondent has been “bothered” in the past month by each of the *DSM-IV-TR* symptoms of PTSD on a scale of 1 (Not at all) to 5 (Extremely). The resulting total score ranges from 17 to 85. A score of at least 50 has frequently been applied in the literature to infer substantial PTSD-symptom burden (+PTSD) and can be considered a relatively conservative threshold with high specificity (35–39), including in this study. Participants scoring below 50 were classified as negative for substantial PTSD symptoms (–PTSD). Internal consistency (coefficient *α*) for the PCL-M has been reported as equaling 0.75 or better; test–retest reliability after 2–3 days exceeds 0.70 (40–42).

The Personality Assessment Inventory (43) is a 344-item, multi-scale, self-report measure of personality and psychopathology. The PAI contains four primary symptom-validity scales, all of which were used with the M-FAST to exclude invalid self-reporters per skyline cutoffs indicated in the manual. Along with validity scales, the PAI contains numerous substantive scales assessing clinical and treatment-relevant considerations. Traumatic Stress (ART-T) is an eight-item subscale of Anxiety Related Disorders and assesses ongoing symptoms and distress as related to a prior traumatic event. This scale was used in conjunction with the PCL-M to identify those with significant symptoms of PTSD, in part as the *DSM-5-TR* criteria are different than the *DSM-IV* criteria upon which the PCL-M is based; by including ARD-T, comparisons can proceed and differences mitigated. A cutoff score of T ≥ 70 was used per manual recommendation.

### Analyses

Data were analyzed using SAS Enterprise Guide (SAS Institute Inc., Cary, NC, USA). The alpha level for statistical significance was established at *p* < 0.05. Total scores on the UPSIT were not normally distributed (*W* = 0.84, *p* < 0.001, skew = −2.02, kurtosis = 6.86); nonparametric alternatives were thus used for analyses. Additionally, because olfaction performance declines with age, we examined whether a significant relationship between the UPSIT score and age was present in our sample to determine whether age was required as a covariate; however, age did not significantly correlate with the UPSIT total score, *ρ* = 0.02, *p* = 0.755. For H1, Mann–Whitney U tests were used when comparing the two groups +TBI versus –TBI. Kruskal–Wallis tests were used for analyses for H2 evaluating TBI severity, as well as with exploratory analyses related to number of mTBIs. Olfactory differences across those with and without significant PTSD symptoms were evaluated also using Mann–Whitney U tests (H3). For H4, we evaluated any additive effects of PTSD above TBI by first using a Kruskal–Wallis test across four groupings (+TBI, +PTSD, +TBI/+PTSD, –TBI/–PTSD), followed by evaluation of PTSD within each TBI-severity group using Mann–Whitney U tests. *Post-hoc* tests for all Kruskal–Wallis tests were analyzed using the Dwass, Steel, Critchlow–Fligner procedure for multiple comparisons.

## Results

Table 2 presents descriptive statistics for UPSIT scores across all subgroups. UPSIT total scores were compared across the subsample with a history of any TBI (+TBI; *n* = 105) and those without a history of TBI (–TBI; *n* = 171). Results (H1) of the Mann–Whitney U were significant but with a small effect, *U* = 13,017, *z* = −2.38, *p* = 0.017, *r* = 0.14. *Post-hoc* power was calculated to equal 60.2%. For H2 evaluating olfaction and TBI severity, the Kruskal–Wallis test was significant: –TBI *n* = 171, mTBI *n* = 69, moderate/severe TBI *n* = 36, *χ*^2^ (2, *n* = 276) = 6.86, *p* = 0.032. Although none of the *post-hoc* tests yielded significance, the observed effect was driven by the difference between the control and moderate/severe TBI groups (*p* = 0.054). As an exploratory analysis, number of mTBI injuries was examined after splitting the subsample into 0 injuries, 1 injury, and 2 or more injuries to determine whether cumulative injuries were related to poorer olfaction. No association was observed between the UPSIT and number of mTBI injuries, Kruskal–Wallis *χ*^2^ (2, *n* = 233) = 3.24, *p* = 0.198.

**Table 2:** UPSIT scores by diagnostic groupings (*N* = 276).

Diagnostic group	*N*	*M*	Median	SD	Min–max	Skewness	Kurtosis
Control	122	33.90	35.00	4.07	18–40	−1.73	3.64
PTSD burden (+)	107	33.55	35.00	4.80	6–40	−2.56	10.42
PTSD burden (−)	169	33.53	34.00	4.10	18–40	−1.47	2.57
No TBI	171	34.02	35.00	3.93	18–40	−1.65	3.56
TBI any severity	105	32.76	33.00	4.94	6–39	−2.22	8.06
Mild TBI	69	33.28	34.00	4.66	6–39	−3.29	16.89
Moderate/severe TBI	36	31.78	33.00	5.36	19–39	−0.89	0.07

UPSIT, University of Pennsylvania Smell Identification Test; PTSD, posttraumatic stress disorder; TBI, traumatic brain injury.

With respect to PTSD symptoms, the two measures of PTSD were not significantly correlated with the UPSIT total score: PCL-M *r* = −0.01, *p* = 0.919; ARD-T *r* = 0.02, *p* = 0.786. To further explore PTSD symptoms, correlations examined between the PCL-M cluster totals and the UPSIT were all nonsignificant: cluster B *r* = −0.06, *p* = 0.377; cluster C *r* = −0.02, *p* = 0.750; cluster D *r* = 0.02, *p* = 0.732. For H3, olfaction was compared between the +PTSD and –PTSD groups: results of the Mann–Whitney U analysis were not significant, *U* = 15092.50, *z* = 0.42, *p* = 0.672, *r* = 0.03. *Post-hoc* power was calculated to equal 2.7%. Results of H4 evaluating whether PTSD symptoms were additive beyond TBI were not significant across TBI and PTSD groups: –TBI/–PTSD *n* = 122, –TBI/+PTSD *n* = 49, +TBI/–PTSD *n* = 47, +TBI/+PTSD *n* = 58, *χ*^2^ (3, *n* = 276) = 7.45, *p* = 0.059. None of the *post-hoc* analyses were significant; specifically, the +TBI/+PTSD group did not significantly differ from the +TBI/–PTSD group (*p* = 0.570), indicating that PTSD symptom burden was not associated with incremental olfaction deficits above and beyond TBI alone. Finally, Table 3 shows results of Mann–Whitney U tests comparing UPSIT scores for +PTSD to –PTSD with groups of no TBI, mTBI, and moderate/severe TBI. None of these tests were significant.

**Table 3:** UPSIT scores for PTSD symptom burden by TBI groups.

TBI	PTSD	Group	*N*	*U*	*z*	*p*	*R*
None	No	–TBI/–PTSD	122	4320.00	0.36	0.359	0.03
	Yes	–TBI/+PTSD	49				
Mild	No	+TBI/–PTSD	34	1101.00	−1.07	0.143	0.13
	Yes	+TBI/+PTSD	35				
Moderate/severe	No	+TBI/–PTSD	13	207.00	−1.09	0.138	0.18
	Yes	+TBI/+PTSD	23				

UPSIT, University of Pennsylvania Smell Identification Test; PTSD, posttraumatic stress disorder; TBI, traumatic brain injury.

## Discussion

Both physical and psychological trauma were experienced by military personnel deployed during the OEF/OIF service era. Due to the consequent neurological and other health effects, psychiatric dysfunction, and putative association of both TBI and PTSD with the subsequent declaration of neurodegenerative disorders (2, 38, 39, 44–51), VA has encountered and will continue to see substantial healthcare utilization and fiscal expenditures arising from this cohort (52). Refinement of differential diagnosis and specification of the comorbid effects of TBI and PTSD may advance more efficacious treatment and enhance veteran quality of life. Results of the current study, the largest to our knowledge to examine olfaction in post-deployed military personnel, support the assertion that olfactory testing may be helpful in the assessment of those with a history of TBI, especially to inform rehabilitation efforts, but that PTSD is not an additive confound to that effect.

Examination of multiple-choice identification of odorants for participants with documented and self-reported TBI history offers support for our first hypothesis in revealing significantly poorer performance for those in the +TBI group compared with the –TBI group. Findings indicate that the statistically significant, but small, effect was driven by the difference between the control –TBI and moderate/severe TBI groups (*p* = 0.054). *Post-hoc* analyses did not reveal differences between the control –TBI and mTBI group. A potential explanation for this finding is that moderate/severe TBI would reasonably be expected to result in greater externally applied force to the head-and-neck area, thereby resulting in greater physical disruption of the neuroanatomical structures and pathways associated with the olfactory bulb. The olfactory bulb lies in a neuroanatomical area of established vulnerability to applied external forces (53). These results are consistent with a prior study on blast-specific TBI in a sample of veterans, as normosmia was found in the mTBI participants, and olfactory impairment was found in some moderate/severe TBI patients, particularly those with frontal-lobe injury (54).

Extant literature in the civilian and military population is mixed across the TBI-severity spectrum in terms of findings of posttraumatic olfactory dysfunction. Variation in findings may be attributed to time since injury, injury severity, injury characteristics, and method of quantifying impairment (18). In mTBI among athletes, for example, no differences in olfaction were reported following acute injury; however, longer elapsed time since the most recent concussion was associated with significantly worse olfaction (55). In one recent cross-sectional study of military veterans, while those with mTBI history self-endorsed olfactory disturbance, no differences were observed in objective olfactory discrimination relative to controls. The authors attributed the observed self-reported olfactory disturbances to the potential presence of emotional distress rather than direct consequences of mTBI (56). Longer durations of LOC in moderate/severe TBI may reflect more severe and global injury, including to the frontal lobes, which are more susceptible to the typical direction of impact and where a substantial portion of the olfactory network resides (5, 7, 8, 23, 57–60). Ogawa and associates (7), in a sample of 365 individuals with occupationally acquired TBI, observed that reduced olfactory sensitivity and/or identification exhibited a significantly higher association with LOC greater than 1 h compared with LOC less than 1 h or absent. In another retrospective study of 68 consecutive patients admitted to a brain-rehabilitation program, Callahan and colleagues (25) reported that those exhibiting partial or total anosmia spent significantly more days in a coma than patients with normosmia (25). Levin et al. (61), using the Olfactory Identification Test in 52 patients, found significantly decreased olfactory-naming and -recognition performance in relation to duration of coma from less than 24 h, to 1–21 days, to more than 21 days.

The present results are partially consistent with prior literature suggesting that increased duration of LOC, combined with impaired olfactory identification, enhance confidence in a diagnostic inference of positive TBI history. Future research is indicated to further elucidate how emotional processing may affect the identification and discrimination of specific odorants. Additionally, given the putative roles of the orbitofrontal cortex, medial prefrontal cortex, temporal lobes, hippocampus, and amygdala in both PTSD and the mediation of smell memory, future investigations should consider brain structure in olfactory studies within military-TBI populations (62). Future studies may also examine the associations between olfactory deficits, immediate posttraumatic neurological sequelae, and differential implications for the prediction of specific psychiatric and behavioral presentations in veterans post TBI (19), as most prior work has addressed civilians (7, 12, 18, 25, 63–67).

A separate inquiry into whether the number of mTBI injuries resulted in greater impairment in olfactory discrimination revealed no differences between groups. While additional exploration is warranted given the high numbers of OEF/OIF personnel with history of multiple concussive injuries, our findings suggest TBI of moderate to severe severity, more than multiple mild concussive impacts, can be associated with olfactory-functioning deficits. These results offer mixed support for our second hypothesis that olfaction performance would be negatively associated with severity of TBI history. An important caveat and potential limitation is the importance of the rigor of TBI severity assignment, especially given emerging data suggestive of a myriad of chronic and potentially disabling symptoms secondary to exposure to multiple subconcussive events (68).

Our findings did not support our third or fourth hypotheses that poorer olfaction performance would be associated with the presence of PTSD symptomatology or that PTSD would have a synergistic effect on olfactory dysfunction above TBI. Our findings stand in contrast to the studies of Vasterling et al. (21) and Dileo et al. (19) in that we did not observe a significant difference between participants with and without substantial PTSD-symptom burden. Vasterling et al. (21) reported a marginally significant correlation (*r* = −0.21) between UPSIT scores and PTSD severity assessed with the Mississippi Scale. In contrast to our OEF/OIF-era active-duty and veteran sample, participants in the two prior studies noting olfactory impairment were Vietnam War veterans, older, and from a different service era, with potential exposure to the toxic defoliant dioxin (Agent Orange), which might have affected the nasal epithelium (21, 69). In contrast to Agent Orange exposures of the Vietnam-era cohort, OEF/OIF veterans experienced different exposures, notably with burn pits and other toxins; thus, if chemical exposure drove the effect in the Vietnam sample, a similar effect might be expected with OEF/OIF samples. Similarly, Vasterling and colleagues (20) in a subsequent study examining Persian Gulf War veterans, who also encountered substantial toxic exposures, failed to find a relationship between UPSIT scores and PTSD severity on an updated version of the Mississippi Scale, and Ruff et al. (17) reported a non-significant correlation between the Brief Smell Identification Test and PCL-M in veterans with a history of TBI from the Afghanistan and Iraq wars. Future research might further explore whether specific smells are related to PTSD, as opposed to only global olfactory ability (19).

Despite current study strengths that included a representation from all military service branches, methodological limitations existed, including case assignment and other statistical issues. First, the method of historical TBI identification is not considered gold standard relative to more recent literature and suggested practice guidelines (70–72). Additionally, the LOC threshold for mTBI used in the measure is 20 min or less, which is not fully consistent with the current VA/DoD criteria of LOC 30 min or less. While participants from the Polytrauma rehabilitation sample had documented TBIs, most injuries for the whole study sample were identified through a self-report screening measure, often years post injury. Retrospective recollection can be distorted by ongoing psychiatric presentations or perceptions of impairment, leading to misattribution or overreporting (73). The Ivins TBI Screen was also limited by a cap on the number of TBIs that could be reported. A response of “Cannot Recall” resulted in assignment to the –TBI group, which lent a degree of uncertainty to the analyses examining number of TBIs.

Second, while two measures were utilized to determine presence or absence of PTSD symptom burden (PCL-M and PAI) and a validity score cut-off was employed, other more-established “gold standard” structured-interview techniques exist to determine PTSD diagnosis (e.g., Clinician-Administered PTSD Scale or Structured Clinical Interview for DSM). Also of note is that due to the date range of the current study, *DSM-IV-TR* was utilized instead of the more recent *DSM-5* diagnostic manual. Relatedly, additional participant data such as psychiatric comorbidities were not available to characterize. Another limitation is that a wide range of durations existed between testing and the most-recent reported TBI, although analysis of TBIs incurred multiple years prior to study represents a common practice in this literature (4, 18, 25, 26). Additionally, both primary analyses were underpowered based on *post-hoc* power analyses, a limitation especially true for analyses using TBI severity, with the smallest subgroup (moderate/severe +TBI/–PTSD) only containing 13 subjects. Thus, conclusions are tentative, and further investigation with larger sample sizes is warranted. Finally, there were demographic differences across the +TBI and –TBI groups (age, race, education) and the PTSD and no PTSD groups (race and education). A larger sample would better allow exploration of those differences.

Future studies might additionally enhance sensitivity to the detection of olfactory alterations by incorporating an examination of laterality (e.g., via monorhinal administration or double simultaneous stimulation) (74, 75). Prior studies have reported a right-nostril advantage in odor discrimination (76, 77), and the right but not the left hippocampus has been noted to show activation during odor identification in an fMRI paradigm (78). Evidence furthermore suggests that the two cerebral hemispheres process different olfactory percepts and that connectivity between the two sides integrates perception (79, 80). For instance, the right orbitofrontal cortex has been reported to be more sensitive to pleasant odors and the left homolog to unpleasant odors (81). PTSD patients suffering from negative cognitions and mood might therefore be expected to display greater activation of left-hemisphere olfactory regions on PET or fMRI in comparison with the right hemisphere. Probing of laterality in future olfactory investigations of PTSD, including assessment of cranial nerve V contralateral innervation, may refine the capacity to localize neuroanatomical alterations and thereby enhance diagnostic precision and inform treatment interventions.

Despite the foregoing limitations, our results indicate that a brief evaluation of olfactory-identification abilities is possibly a useful outcome in clinical and research investigations of TBI. Additional research is required to further understand the relationship between TBI severity and olfactory disfunction. Investigation of other indices and subcomponents of olfactory-system processing and the role of laterality, coupled with neuroimaging, may further assist in differentiating the neuropsychological manifestations of physical and psychological trauma in veterans and other populations.

## Data Availability

The datasets presented in this article are available to qualified investigators upon reasonable request. Requests to access the datasets should be directed to RS, robert.shura2@va.gov.

## Author’s note

The views, opinions, and/or findings contained in this article are those of the authors and should not be construed as an official VA or DoD position, policy, or decision unless so designated by other official documentation. Additionally, claims expressed in this article do not necessarily represent those of their affiliated organizations, or those of the publisher, the editors and the reviewers. Any product that may be evaluated in this article, or claim that may be made by its manufacturer, is not guaranteed or endorsed by the publisher.

## References

[1] Warden D. Military TBI during the Iraq and Afghanistan wars. J Head Trauma Rehabil. (2006) 21:398–402. doi: 10.1097/00001199-200609000-00004, PMID: 16983225

[2] Weiner MW Friedl KE Pacifico A Chapman JC Jaffee MS Little DM. Military risk factors for Alzheimer's disease. Alzheimers Dement. (2013) 9:445–51. doi: 10.1016/j.jalz.2013.03.005, PMID: 23809365 PMC5904389

[3] Walker WC Franke LM McDonald SD Sima AP Keyser-Marcus L. Prevalence of mental health conditions after military blast exposure, their co-occurrence, and their relation to mild traumatic brain injury. Brain Inj. (2015) 29:1581–8. doi: 10.3109/02699052.2015.1075151, PMID: 26479126

[4] Petrie EC Cross DJ Yarnykh VL Richards T Martin NM Pagulayan K. Neuroimaging, behavioral, and psychological sequelae of repetitive combined blast/impact mild traumatic brain injury in Iraq and Afghanistan war veterans. J Neurotrauma. (2014) 31:425–36. doi: 10.1089/neu.2013.2952, PMID: 24102309 PMC3934596

[5] Doty RL Doty RL. A review of olfactory dysfunctions in man. Am J Otolaryngol. (1979) 1:57–79. doi: 10.1016/s0196-0709(79)80010-1, PMID: 399716

[6] Nordin S Murphy C Davidson TM Quinonez C Jalowayski AA Ellison DW. Prevalence and assessment of qualitative olfactory dysfunction in different age groups. Laryngoscope. (1996) 106:739–44. doi: 10.1097/00005537-199606000-00014, PMID: 8656960

[7] Ogawa T Rutka J. Olfactory dysfunction in head injured workers. Acta Otolaryngol. (1999): 50–7. doi: 10.1080/00016489950181206 10445080

[8] Eslinger PJ Damasio AR Van Hoesen GW. Olfactory dysfunction in man: anatomical and behavioral aspects. Brain Cogn. (1982) 1:259–85. doi: 10.1016/0278-2626(82)90028-8, PMID: 6765474

[9] Wang J Eslinger PJ Smith MB Yang QX. Functional magnetic resonance imaging study of human olfaction and normal aging. J Gerontol A Biol Sci Med Sci. (2005) 60:510–4. doi: 10.1093/gerona/60.4.510, PMID: 15933393

[10] McNeill E Ramakrishnan Y Carrie S. Diagnosis and management of olfactory disorders: survey of UK-based consultants and literature review. J Laryngol Otol. (2007) 121:713–20. doi: 10.1017/S0022215107006615, PMID: 17359559

[11] Hoffman HJ Cruickshanks KJ Davis B. Perspectives on population-based epidemiological studies of olfactory and taste impairment. Ann N Y Acad Sci. (2009) 1170:514–30. doi: 10.1111/j.1749-6632.2009.04597.x, PMID: 19686188 PMC2760342

[12] Proskynitopoulos PJ Stippler M Kasper EM. Post-traumatic anosmia in patients with mild traumatic brain injury (mTBI): a systematic and illustrated review. Surg Neurol Int. (2016) 7:S263–75. doi: 10.4103/2152-7806.181981, PMID: 27213113 PMC4866055

[13] Burstein A. Olfactory hallucinations. Hosp Community Psych. (1987) 38:80. doi: 10.1176/ps.38.1.80, PMID: 3804247

[14] Yousem DM Geckle RJ Bilker WB Kroger H Doty RL. Posttraumatic smell loss: relationship of psychophysical tests and volumes of the olfactory bulbs and tracts and the temporal lobes. Acad Radiol. (1999) 6:264–72. doi: 10.1016/S1076-6332(99)80449-8, PMID: 10228615

[15] Reden J Mueller A Mueller C Konstantinidis I Frasnelli J Landis BN. Recovery of olfactory function following closed head injury or infections of the upper respiratory tract. Arch Otolaryngol Head Neck Surg. (2006) 132:265–9. doi: 10.1001/archotol.132.3.265, PMID: 16549746

[16] Haxel BR Grant L Mackay-Sim A. Olfactory dysfunction after head injury. J Head Trauma Rehabil. (2008) 23:407–13. doi: 10.1097/01.HTR.0000341437.59627.ec, PMID: 19033834

[17] Ruff RL Riechers RG II Wang X-F Piero T Ruff SR. A case-control study examining whether neurological deficits and PTSD in combat veterans are related to episodes of mild TBI. BMJ Open. (2011) 2:1–12. doi: 10.1136/bmjopen-2011-000312 PMC3312078 22431700

[18] Schofield PW Moore MM Gardner A. Traumatic brain injury and olfaction: a systematic review. Front Neurol. (2014) 5:1–22. 24478752 10.3389/fneur.2014.00005 PMC3897870

[19] Dileo JF Brewer WJ Hopwood M Anderson V Creamer M. Olfactory identification dysfunction, aggression and impulsivity in war veterans with post-traumatic stress disorder. Psychol Med. (2008) 38:523–31. doi: 10.1017/S0033291707001456, PMID: 17903334

[20] Vasterling JJ Brailey K Tomlin H Rice J Sutker PB. Olfactory functioning in Gulf War-era veterans: relationships to war-zone duty, self-reported hazards exposures, and psychological distress. J Int Neuropsychol Soc. (2003) 9:407–18. doi: 10.1017/S1355617703930062, PMID: 12666765

[21] Vasterling JJ Brailey K Sutker PB. Olfactory identification in combat-related posttraumatic stress disorder. J Trauma Stress. (2000) 13:241–53. doi: 10.1023/A:1007754611030, PMID: 10838673

[22] Doty RL Shaman P Dann M. Development of the University of Pennsylvania Smell Identification Test: a standardized microencapsulated test of olfactory function. Physiol Behav. (1984) 32:489–502. doi: 10.1016/0031-9384(84)90269-5, PMID: 6463130

[23] Yousem DM Geckle RJ Bilker WB McKeown DA Doty RL. Posttraumatic olfactory dysfunction: MR and clinical evaluation. AJNR Am J Neuroradiol. (1996) 17:1171–9. 8791933 PMC8338600

[24] Zald DH Pardo JV. Emotion, olfaction, and the human amygdala: amygdala activation during aversive olfactory stimulation. Proc Natl Acad Sci USA. (1997) 94:4119–24. doi: 10.1073/pnas.94.8.4119, PMID: 9108115 PMC20578

[25] Callahan CD Hinkebein J. Neuropsychological significance of anosmia following traumatic brain injury. J Head Trauma Rehabil. (1999) 14:581–7. doi: 10.1097/00001199-199912000-00006, PMID: 10671703

[26] Callahan CD Hinkebein JH. Assessment of anosmia after traumatic brain injury: performance characteristics of the University of Pennsylvania Smell Identification Test. J Head Trauma Rehabil. (2002) 17:251–6. doi: 10.1097/00001199-200206000-00006, PMID: 12086578

[27] Fortin A Lefebvre MB Ptito M. Traumatic brain injury and olfactory deficits: the tale of two smell tests! Brain Inj. (2010) 24:27–33. doi: 10.3109/02699050903446815, PMID: 20001480

[28] Brancu M Wagner HR Morey RA Beckham JC Calhoun PS Tupler LA. The post-deployment mental health (PDMH) study and repository: a multi-site study of US Afghanistan and Iraq era veterans. Int J Methods Psychiatr Res. (2017) 26:e1570. doi: 10.1002/mpr.1570 28656593 PMC6492939

[29] Doty RL. The Smell Identification Test administration manual. 3rd ed. Haddon Heights, NJ: Sensonics, Inc. (1995).

[30] Doty RL Frye RE Agrawal U. Internal consistency reliability of the fractionated and whole University of Pennsylvania Smell Identification Test. Percept Psychophys. (1989) 45:381–4. doi: 10.3758/BF03210709, PMID: 2726398

[31] Doty RL Newhouse MG Azzalina JD. Internal consistency and short-term test-retest reliability of the University of Pennsylvania Smell Test. Chem Senses. (1985) 10:297–300.

[32] Ivins BJ Schwab KA Warden D Harvey LTCS Hoilien MAJM Powell COLJ. Traumatic brain injury in U.S. Army paratroopers: prevalence and character. J Trauma. (2003) 55:617–21. doi: 10.1097/01.TA.0000052368.97573.D4, PMID: 14566111

[33] Green P. Green's Word Memory Test for Microsoft Windows: user manual. Rev. ed. 2005 ed. Edmonton: Green's Publications Inc. (2005).

[34] Miller HA. M-FAST: Miller Forensic Assessment of Symptoms Test: professional manual. Odessa, FL: Psychological Assessment Resources (2001).

[35] McDonald SD Calhoun PS. The diagnostic accuracy of the PTSD Checklist: a critical review. Clin Psychol Rev. (2010) 30:976–87. doi: 10.1016/j.cpr.2010.06.012, PMID: 20705376

[36] Walker WC McDonald SD Franke LM. Diagnostic accuracy of Posttraumatic Stress Disorder Checklist in blast-exposed military personnel. J Rehabil Res Dev. (2014) 51:1203–16. doi: 10.1682/JRRD.2013.12.0271, PMID: 25671462

[37] Tsai J Pietrzak RH Hoff RA Harpaz-Rotem I. Accuracy of screening for posttraumatic stress disorder in specialty mental health clinics in the U.S. Veterans Affairs healthcare system. Psychiatry Res. (2016) 240:157–62. doi: 10.1016/j.psychres.2016.04.036, PMID: 27107669

[38] Erbes C Westermeyer J Engdahl B Johnsen E. Post-traumatic stress disorder and service utilization in a sample of service members from Iraq and Afghanistan. Mil Med. (2007) 172:359–63. doi: 10.7205/MILMED.172.4.359, PMID: 17484303

[39] McDevitt-Murphy ME Williams JL Bracken KL Fields JA Monahan CJ Murphy JG. PTSD symptoms, hazardous drinking, and health functioning among U.S. OEF and OIF veterans presenting to primary care. J Trauma Stress. (2010) 23:108–11. doi: 10.1002/jts.20482, PMID: 20104586 PMC2876344

[40] Wilkins KC Lang AJ Norman SB. Synthesis of the psychometric properties of the PTSD Checklist (PCL) military, civilian, and specific versions. Depress Anxiety. (2011) 28:596–606. doi: 10.1002/da.20837, PMID: 21681864 PMC3128669

[41] Bjornestad AG Schweinle A Elhai JD. Measuring secondary traumatic stress symptoms in military spouses with the Posttraumatic Stress Disorder Checklist Military version. J Nerv Ment Dis. (2014) 202:864–9. doi: 10.1097/NMD.0000000000000213, PMID: 25386765

[42] Armour C Contractor A Elhai JD Stringer M Lyle G Forbes D. Identifying latent profiles of posttraumatic stress and major depression symptoms in Canadian veterans: exploring differences across profiles in health related functioning. Psychiatry Res. (2015) 228:1–7. doi: 10.1016/j.psychres.2015.03.011, PMID: 25936834

[43] Morey LC Ambwani S. The personality assessment inventory In: The SAGE handbook of personality theory and assessment, Vol 2: Personality measurement and testing. Thousand Oaks, CA, US: Sage Publications, Inc. (2008). 626–45.

[44] Goldman SM Tanner CM Oakes D Bhudhikanok GS Gupta A Langston JW. Head injury and Parkinson's disease risk in twins. Ann Neurol. (2006) 60:65–72. doi: 10.1002/ana.20882, PMID: 16718702

[45] Chen H Richard M Sandler DP Umbach DM Kamel F. Head injury and amyotrophic lateral sclerosis. Am J Epidemiol. (2007) 166:810–6. doi: 10.1093/aje/kwm153, PMID: 17641152 PMC2239342

[46] Gavett BE Stern RA Cantu RC Nowinski CJ McKee AC. Mild traumatic brain injury: a risk factor for neurodegeneration. Alzheimer's Res Ther. (2010) 2:18–8. doi: 10.1186/alzrt42, PMID: 20587081 PMC2919698

[47] Yaffe K Vittinghoff E Lindquist K Barnes D Covinsky KE Neylan T. Posttraumatic stress disorder and risk of dementia among US veterans. Arch Gen Psychiatry. (2010) 67:608–13. doi: 10.1001/archgenpsychiatry.2010.61, PMID: 20530010 PMC2933793

[48] Qureshi SU Kimbrell T Pyne JM Magruder KM Hudson TJ Petersen NJ. Greater prevalence and incidence of dementia in older veterans with posttraumatic stress disorder. J Am Geriatr Soc. (2010) 58:1627–33. doi: 10.1111/j.1532-5415.2010.02977.x, PMID: 20863321

[49] Sivanandam TM Thakur MK. Traumatic brain injury: a risk factor for Alzheimer's disease. Neurosci Biobehav Rev. (2012) 36:1376–81. doi: 10.1016/j.neubiorev.2012.02.013, PMID: 22390915

[50] Weiner MW Veitch DP Hayes J Neylan T Grafman J Aisen PS. Effects of traumatic brain injury and posttraumatic stress disorder on Alzheimer's disease in veterans, using the Alzheimer's Disease Neuroimaging Initiative. Alzheimers Dement. (2014) 10:S226–35. doi: 10.1016/j.jalz.2014.04.005, PMID: 24924673 PMC4392759

[51] Meziab O Kirby KA Williams B Yaffe K Byers AL Barnes DE. Prisoner of war status, posttraumatic stress disorder, and dementia in older veterans. Alzheimers Dement. (2014) 10:S236–41. doi: 10.1016/j.jalz.2014.04.004, PMID: 24924674

[52] Geiling J Rosen JM Edwards RD. Medical costs of war in 2035: long-term care challenges for veterans of Iraq and Afghanistan. Mil Med. (2012) 177:1235–44. doi: 10.7205/MILMED-D-12-00031, PMID: 23198496

[53] Han P Winkler N Hummel C Hahner A Gerber J Hummel T. Alterations of brain gray matter density and olfactory bulb volume in patients with olfactory loss after traumatic brain injury. J Neurotrauma. (2018) 35:2632–40. doi: 10.1089/neu.2017.5393, PMID: 29699465

[54] Xydakis MS Mulligan LP Smith AB Olsen CH Lyon DM Belluscio L. Olfactory impairment and traumatic brain injury in blast-injured combat troops: a cohort study. Neurology. (2015) 84:1559–67. doi: 10.1212/WNL.0000000000001475, PMID: 25788559 PMC4408285

[55] Charland-Verville V Lassonde M Frasnelli J. Olfaction in athletes with concussion. Am J Rhinol Allergy. (2012) 26:222–6. doi: 10.2500/ajra.2012.26.3769, PMID: 22643951

[56] Rothman DJ McDonald SD Walker WC Feldman G. Olfactory changes after military deployment are associated with emotional distress but not with mild traumatic brain injury history. Am J Phys Med Rehabil. (2022) 101:423–8. doi: 10.1097/PHM.0000000000001839, PMID: 35444152

[57] Sumner D. Post-traumatic anosmia. Brain. (1964) 87:107–20. doi: 10.1093/brain/87.1.107, PMID: 14156077

[58] Schurr PH Schurr PH. Aberrations of the sense of smell in head injury and cerebral tumours. Proc R Soc Med. (1975) 68:470–2. doi: 10.1177/003591577506800802, PMID: 1202480 PMC1863832

[59] Swann IJ Bauza-Rodriguez B Currans R Riley J Shukla V. The significance of post-traumatic amnesia as a risk factor in the development of olfactory dysfunction following head injury. Emerg Med J. (2006) 23:618–21. doi: 10.1136/emj.2005.029017, PMID: 16858094 PMC2564164

[60] McGlade E Rogowska J Yurgelun-Todd D. Sex differences in orbitofrontal connectivity in male and female veterans with TBI. Brain Imaging Behav. (2015) 9:535–49. doi: 10.1007/s11682-015-9379-3, PMID: 25864195 PMC4575683

[61] Levin HS High WM Eisenberg HM. Impairment of olfactory recognition after closed head injury. Brain. (1985) 108:579–91. doi: 10.1093/brain/108.3.579 4041775

[62] Daniels JK Vermetten E. Odor-induced recall of emotional memories in PTSD-review and new paradigm for research. Exp Neurol. (2016) 284:168–80. doi: 10.1016/j.expneurol.2016.08.001 27511295

[63] Varney NR Varney NR. Prognostic significance of anosmia in patients with closed-head trauma. J Clin Exp Neuropsychol. (1988) 10:250–4. doi: 10.1080/01688638808408239, PMID: 3350923

[64] Correia S Faust D Doty RL. A re-examination of the rate of vocational dysfunction among patients with anosmia and mild to moderate closed head injury. Arch Clin Neuropsychol. (2001) 16:477–88. doi: 10.1093/arclin/16.5.477, PMID: 14590161

[65] Greiffenstein FM John Baker W Gola T. Brief report: anosmia and remote outcome in closed head injury. J Clin Exp Neuropsychol. (2002) 24:705–9. doi: 10.1076/jcen.24.5.705.1011, PMID: 12187453

[66] Crowe SF Crowe LM. Does the presence of posttraumatic anosmia mean that you will be disinhibited? J Clin Exp Neuropsychol. (2013) 35:298–308. doi: 10.1080/13803395.2013.771616, PMID: 23432111

[67] Sigurdardottir S Andelic N Skandsen T Anke A Roe C Holthe OO. Olfactory identification and its relationship to executive functions, memory, and disability one year after severe traumatic brain injury. Neuropsychology. (2016) 30:98–108. doi: 10.1037/neu0000206, PMID: 26076319

[68] Bailie JM Lippa SM Hungerford L French LM Brickell TA Lange RT. Cumulative blast exposure during a military career negatively impacts recovery from traumatic brain injury. J Neurotrauma. (2024) 41:604–12. doi: 10.1089/neu.2022.0192, PMID: 37675903

[69] Sycheva LP Umnova NV Kovalenko MA Zhurkov VS Shelepchikov AA Roumak VS. Dioxins and cytogenetic status of villagers after 40 years of agent Orange application in Vietnam. Chemosphere. (2016) 144:1415–20. doi: 10.1016/j.chemosphere.2015.10.009, PMID: 26495825

[70] Corrigan JD Bogner J. Initial reliability and validity of the Ohio State University TBI Identification Method. J Head Trauma Rehabil. (2007) 22:318–29. doi: 10.1097/01.HTR.0000300227.67748.77, PMID: 18025964

[71] Lippa SM Yeh PH Gill J French LM Brickell TA Lange RT. Plasma tau and amyloid are not reliably related to injury characteristics, neuropsychological performance, or white matter integrity in service members with a history of traumatic brain injury. J Neurotrauma. (2019) 36:2190–9. doi: 10.1089/neu.2018.6269, PMID: 30834814 PMC6909749

[72] Walker WC Cifu DX Hudak AM Goldberg G Kunz RD Sima AP. Structured interview for mild traumatic brain injury after military blast: inter-rater agreement and development of diagnostic algorithm. J Neurotrauma. (2015) 32:464–73. doi: 10.1089/neu.2014.3433, PMID: 25264909

[73] Ruff RL Riechers RG 2nd Wang XF Piero T Ruff SS. For veterans with mild traumatic brain injury, improved posttraumatic stress disorder severity and sleep correlated with symptomatic improvement. J Rehabil Res Dev. (2012) 49:1305–20. doi: 10.1682/jrrd.2011.12.0251, PMID: 23408213

[74] Bellas DN Novelly RA Eskenazi B Wasserstein J. The nature of unilateral neglect in the olfactory sensory system. Neuropsychologia. (1988) 26:45–52. doi: 10.1016/0028-3932(88)90029-2, PMID: 3362344

[75] Royet JP Plailly J. Lateralization of olfactory processes. Chem Senses. (2004) 29:731–45. doi: 10.1093/chemse/bjh067 15466819

[76] Zatorre RJ Jones-Gotman M. Right-nostril advantage for discrimination of odors. Percept Psychophys. (1990) 47:526–31. doi: 10.3758/BF03203105, PMID: 2367173

[77] Martinez BA Cain WS de Wijk RA Spencer DD Novelly RA Sass KJ. Olfactory functioning before and after temporal lobe resection for intractable seizures. Neuropsychology. (1993) 7:351–63. doi: 10.1037/0894-4105.7.3.351

[78] Kjelvik G Evensmoen HR Brezova V Håberg AK. The human brain representation of odor identification. J Neurophysiol. (2012) 108:645–57. doi: 10.1152/jn.01036.2010, PMID: 22539820

[79] Dade LA Zatorre RJ Jones-Gotman M. Olfactory learning: convergent findings from lesion and brain imaging studies in humans. Brain. (2002) 125:86–101. doi: 10.1093/brain/awf003, PMID: 11834595

[80] Zatorre RJ Jones-Gotman M Evans AC Meyer E. Functional localization and lateralization of human olfactory cortex. Nature. (1992) 360:339–40. doi: 10.1038/360339a0, PMID: 1448149

[81] Anderson AK Christoff K Stappen I Panitz D Ghahremani DG Glover G. Dissociated neural representations of intensity and valence in human olfaction. Nat Neurosci. (2003) 6:196–202. doi: 10.1038/nn1001, PMID: 12536208

